# PAM50 Subtypes in Baseline and Residual Tumors Following Neoadjuvant Trastuzumab-Based Chemotherapy in HER2-Positive Breast Cancer: A Consecutive-Series From a Single Institution

**DOI:** 10.3389/fonc.2019.00707

**Published:** 2019-08-06

**Authors:** Sonia Pernas, Anna Petit, Fina Climent, Laia Paré, J. Perez-Martin, Luz Ventura, Milana Bergamino, Patricia Galván, Catalina Falo, Idoia Morilla, Adela Fernandez-Ortega, Agostina Stradella, Montse Rey, Amparo Garcia-Tejedor, Miguel Gil-Gil, Aleix Prat

**Affiliations:** ^1^Department of Medical Oncology-Breast Cancer Unit, Institut Català d'Oncologia (ICO)-H.U.Bellvitge-Institut d'Investigació Biomèdica de Bellvitge (IDIBELL), Universitat de Barcelona, Barcelona, Spain; ^2^Department of Pathology-Breast Cancer Unit, Institut Català d'Oncologia (ICO)-H.U.Bellvitge-Institut d'Investigació Biomèdica de Bellvitge (IDIBELL), Universitat de Barcelona, Barcelona, Spain; ^3^Department of Medical Oncology, Hospital Clínic de Barcelona, Universitat de Barcelona, Barcelona, Spain; ^4^Clinical Research Unit, Institut Català d'Oncologia (ICO)-L'Hospitalet, Barcelona, Spain; ^5^Department of Pharmacy, Institut Català d'Oncologia (ICO)-L'Hospitalet, Barcelona, Spain; ^6^Department of Gynecology-Breast Cancer Unit, Institut Català d'Oncologia (ICO)-H.U.Bellvitge-Institut d'Investigació Biomèdica de Bellvitge (IDIBELL), Universitat de Barcelona, Barcelona, Spain

**Keywords:** breast cancer, HER2, pathological complete response, gene expression, molecular intrinsic subtype, residual disease, paired samples

## Abstract

**Introduction:** HER2-enriched subtype has been associated with higher response to neoadjuvant anti-HER2-based therapy across various clinical trials. However, limited data exist in real-world practice and regarding residual disease. Here, we evaluate the association of HER2-enriched with pathological response (pCR) and gene expression changes in pre- and post-treatment paired samples in HER2-positive breast cancer patients treated outside of a clinical trial.

**Methods:** We evaluated clinical-pathological data from a consecutive series of 150 patients with stage II-IIIC HER2-positive breast cancer treated from August 2004 to December 2012 with trastuzumab-based neoadjuvant chemotherapy. Expression of 105 breast cancer-related genes, including the PAM50 genes, was determined in available pre-and post-treatment formalin-fixed paraffin-embedded tumor samples using the nCounter platform. Intrinsic molecular subtypes were determined using the research-based PAM50 predictor. Association of genomic variables with total pCR was performed.

**Results:** The pCR rate was 53.3%, with higher pCR among hormonal receptor (HR)-negative tumors (70 vs. 39%; *P* < 0.001). A total of 89 baseline and 28 residual tumors were profiled, including pre- and post-treatment paired samples from 26 patients not achieving a pCR. HER2-enriched was the predominant baseline subtype not only in the overall and HR-negative cohorts (64 and 75%, respectively), but also in the HR-positive cohort (55%). HER2-enriched was associated with higher pCR rates compared to non-HER2-enriched subtypes (65 vs. 31%; OR = 4.07, 95% CI 1.65–10.61, *P* < 0.002) and this association was independent of HR status. In pre- and post-treatment paired samples from patients not achieving a pCR, a lower proportion of HER2-enriched and twice the number of luminal tumors were observed at baseline, and luminal A was the most frequent subtype in residual tumors. Interestingly, most (81.8%) HER2-enriched tumors changed to non-HER2-enriched, whereas most luminal A samples maintained the same subtype in residual tumors.

**Conclusions:** Outside of a clinical trial, PAM50 HER2-enriched subtype predicts pCR beyond HR status following trastuzumab-based chemotherapy in HER2-positive disease. The clinical value of intrinsic molecular subtype in residual disease warrants further investigation.

## Introduction

Significant advances have occurred in the treatment of HER2-positive breast cancer that have dramatically improved survival and changed its natural history (1–6). In the neoadjuvant setting, the introduction of HER2-targeted agents to chemotherapy has considerably enhanced the achievement of a pathological complete response (pCR) (7–10). This has translated into important gains in survival in early HER2-positive disease (11–13). Despite these improvements, HER2-positive breast cancer remains a clinically and biologically heterogeneous disease with different treatment sensitivities and survival outcomes (14–16). Thus, identification of these distinct groups of patients using molecular-based biomarkers is needed.

Among different molecular biomarkers evaluated to date in HER2-positive disease, intrinsic molecular subtypes (i.e., luminal A, luminal B, HER2-enriched, and basal-like) identified by gene expression analysis have now shown consistent data across several clinical trials. Specifically, the HER2-enriched subtype has been associated with a higher likelihood of achieving a pCR following neoadjuvant anti-HER2-based chemotherapy compared to non-HER2-enriched disease (15, 17–21). However, limited data exist to date (1) outside a clinical trial setting and (2) regarding residual disease and gene expression changes in paired samples.

Based on this prior evidence, the primary aim of this study was to test the association of the HER2-enriched subtype with pCR in a consecutive series of HER2-positive breast cancer patients homogeneously treated with trastuzumab-based neoadjuvant chemotherapy at a single comprehensive cancer center. As a secondary aim, we explored biological changes between baseline and surgery specimens in patients with residual disease after neoadjuvant treatment. Initial clinical results of this series were previously published (22).

## Methods

### Clinical-Pathological Data

Clinicopathological data were evaluated in a series of 150 women with stages II to IIIC (T4d included) HER2-positive breast cancer consecutively treated at Institut Català d'Oncologia (ICO)-Hospitalet (Barcelona, Spain) between August 2004 and December 2012. Treatment schema consisted of weekly paclitaxel 80 mg/m^2^ for 12 weeks followed by 4 cycles of 5-Fluoracil, Epirubicin, and Cyclophosphamide (600/75/60 mg/m^2^) every 21 days. During the 24 weeks of neoadjuvant systemic treatment, concomitant trastuzumab 2 mg/kg (after a 4 mg/kg loading dose) was administered. Surgery was performed 3–4 weeks after the last dose of chemotherapy. Left ventricular ejection fraction was monitored every 12 weeks during treatment and in the follow-up period every 6 months for the first 2 years and then annually. Adjuvant hormonal therapy and radiotherapy were administered per institutional guidelines. Additional 6 months of adjuvant trastuzumab were also recommended since 2006. This study was approved by the Institutional Review Board of H.U. Bellvitge, L'Hospitalet (Barcelona), and all patients signed informed consent forms to allow molecular analyses to be performed on their tissue samples.

Estrogen receptor (ER) and progesterone receptor (PR) status were determined by immunohistochemistry (IHC) at baseline core biopsies and in post-treatment surgical specimens with residual disease and considered positive if >1% of tumor cells were stained. HER2 positivity was determined by IHC and fluorescence *in situ* hybridization according to 2007 ASCO/CAP guidelines (23). pCR was defined as the absence of invasive cancer both in the breast and lymph nodes, regardless of the presence of *in situ* carcinoma (ypT0/isypN0).

### Gene Expression Analysis and Intrinsic Subtyping

Hematoxylin and eosin-stained slides from formalin-fixed paraffin-embedded (FFPE) baseline core biopsies and post-neoadjuvant surgical specimens of patients with residual disease were examined to confirm the presence of invasive tumor cells and to determine the minimum surface area. For RNA purification, 1–5 10 μm FFPE slides were used for each tumor specimen. A minimum of 100 ng of total RNA was used to measure the expression of 105 breast cancer-related genes, including the PAM50 genes, 5 housekeeping genes, and 50 additional genes (related to proliferation, cell cycle, and angiogenesis/hypoxia). Gene expression analyses and comparison of pre- and post-treatment samples were performed at Vall d'Hebron Institute of Oncology (VHIO) using the nCounter platform (Nanostring Technologies, Seattle, WA, USA). Data were log base 2 transformed and normalized using housekeeping genes selected.

Intrinsic subtyping (luminal A, luminal B, HER2-enriched, basal-like, and normal-like) was performed using the research-based PAM50 intrinsic subtype predictor as previously described (24, 25).

### Statistical Analysis

Association between two variables was evaluated using Student's *t*-test, Pearson's χ2 test or Fisher's exact test. Univariate and multivariate logistic regression analyses were done to investigate the association of each variable with pCR. Odds ratios (OR) and 95% confidence intervals (CI) were calculated for each variable. The significance level was set to a two-sided α of 0.05. To identify genes whose expression was significantly different between paired pre- and post-treated samples, we used a paired two-class significance analysis of microarrays (SAM) with a false discovery rate (FDR) <5%. All statistical tests were two sided, and the statistical significance level was set to <0.05. We used R version 3.2.2 for all the statistical analyses (http://cran.r-project.org).

## Results

Baseline clinicopathologic characteristics of the overall cohort of patients (*n* = 150) and from those with tissue samples available for gene expression (*n* = 91) are listed in Table 1. A flow diagram of the study population is shown in Figure S1. The baseline median tumor size was 30 mm and 35% of patients had locally advanced breast cancer. All 150 patients underwent surgery; therefore, all were evaluable for pathological response. Lumpectomy was performed in 87 patients (58%). Overall, 80 of 150 patients (53.3%, 95% CI 0.45–0.61) achieved a pCR in the breast and lymph nodes. Interestingly, 10 patients out of 13 (77%) with inflammatory breast cancer experienced a pCR. HR-negative disease was significantly associated with higher pCR rates (69.6% [48/69] vs. 39.5% [32/81] in HR-positive; *P* < 0.001). Age, tumor size, histological differentiation grade, or Ki67 were not associated with pCR.

**Table 1:** Baseline patient characteristics of the entire cohort and of patients with genomic data.

	**All patients *n* = 150**	**Patients with genomic data *n* = 91**
Age, median (range)	50 (27–79)	51 (27–76)
**HISTOLOGICAL GRADE**		
Grade 2	47 (31.3%)	33 (36.3%)
Grade 3	101 (67.3%)	57 (62.6%)
Not evaluable	2 (1.3%)	1 (1.1%)
**TUMOR STAGE**		
T1	9 (6.0%)	7 (7.8%)
T2	97 (64.7%)	55 (61.1%)
T3	15 (10.0%)	8 (8.9%)
T4b	16 (10.6 %)	10 (11.1%)
T4d	13 (8.7%)	10 (11.1%)
**NODAL STATUS**		
N0	34 (22.5%)	18 (20%)
N1	90 (59.6%)	57 (63.3%)
N2	15 (9.9%)	10 (11.1%)
N3	11 (7.3%)	5 (5.6%)
**HORMONAL RECEPTOR STATUS**		
ER+ PR+	56 (37.4%)	36 (39.6%)
ER+ PR–	22 (14.4%)	11(12.1%)
ER– PR+	2 (1.2%)	2 (2.2%)
ER– PR–	70 (47.0%)	42 (46.1%)
**Ki67**		
<20	27 (18.0%)	18 (19.8%)
≥20	119 (79.3%)	73 (80.2%)
Not evaluable	4 (2.7%)	

*ER, estrogen receptor; PR, progesterone receptor*.

**Figure 1:**
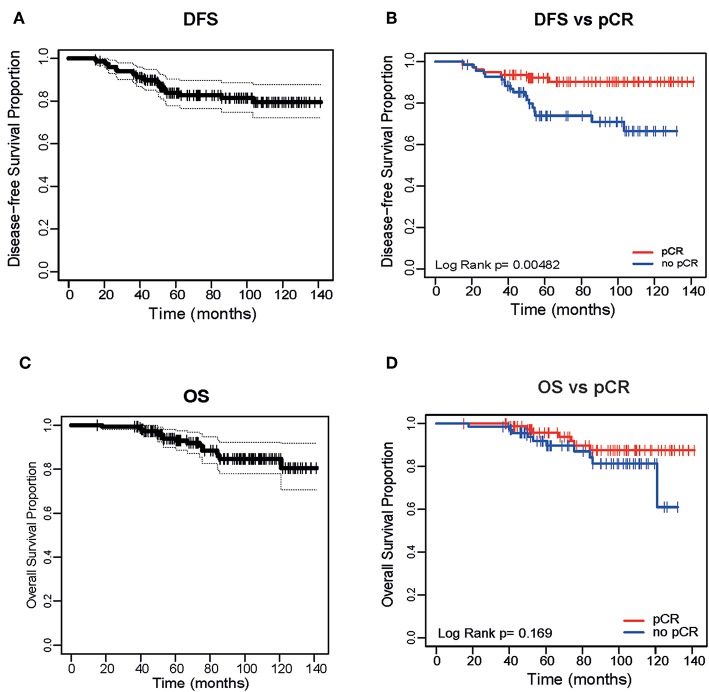
Disease Free Survival of the entire cohort **(A)** and based on pathological complete response (pCR) **(B)**. Overall Survival (OS) of the entire cohort **(C)** and based on pCR **(D)**.

With a median of follow-up of 79 months (range 15–141 months), median disease-free survival (DFS) was not reached (Figure 1A); DFS was 83% (95% CI 72.1–87.6%). There were 25 relapses (16.7%): 16 patients had initially HR-positive tumors and 9 HR-negative tumors. Median time to progression was 32 months (range 8–96 months). This time differed significantly per HR status: 19.8 months in HR-negative tumors and double (39.5 months) in HR-positive ones (*p* = 0.023). Achieving a pCR was significantly associated with an improved DFS in the overall cohort (Figure 1B) and by HR status (Figure S2). There were 7 relapses (8.7%) in the pCR group vs. 18 (25.7%) in the group of residual disease (*p* = 0.005, OR 3.28, 95% CI 1.37–7.86). Median overall survival (OS) was not reached (Figure 1C). OS was 88.7% (95% CI 70.6–91.8%). There were 17 deaths, the majority due to disease progression and 3 due to other causes (none of these related to treatment). In contrast to DFS, achieving a pCR was not significantly associated with an improved OS (Figure 1D).

### Baseline Subtype Distribution

**Figure 2:**
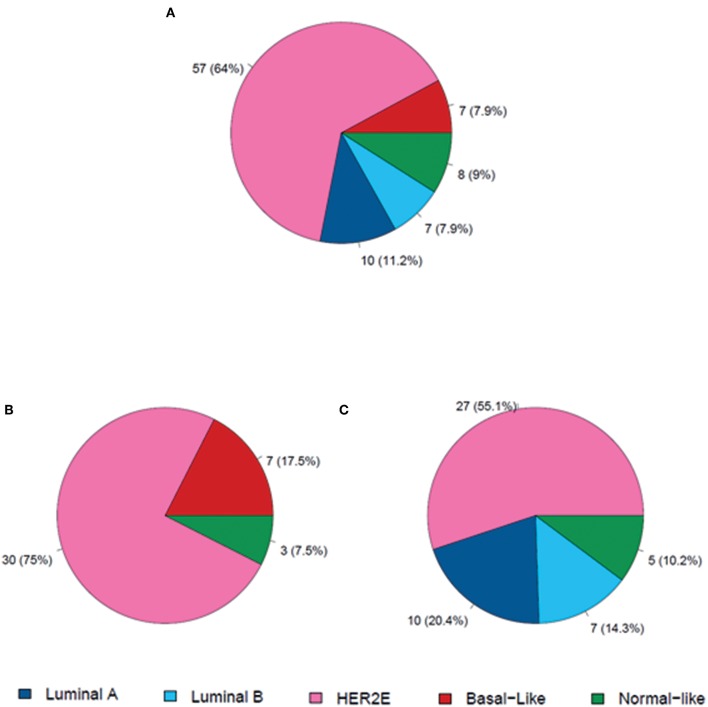
Distribution of molecular subtypes at baseline. **(A)** In all patients (*n* = 89); **(B)** Patients with HR-negative disease (*n* = 40); **(C)** Patients with HR-positive disease (*n* = 49).

Of the 89 available baseline samples for gene expression analyses, 40 were HR-negative and 49 were HR-positive. At baseline, most tumors were classified as HER2-enriched subtype by PAM50 (64%), followed by luminal A (11.2%), normal-like (9%) basal-like (7.9%), and luminal B (7.9%). Subtype distribution differed significantly between HR-status. Basal-like subtype was identified only in HR-negative disease, whereas luminal A and B were identified only in HR-positive samples (Figure 2) HER2-enriched was the predominant one in both HR-negative tumors (75%) and HR-positive tumors (55%).

### Association of Intrinsic Subtypes and Gene Expression With pCR

**Figure 3:**
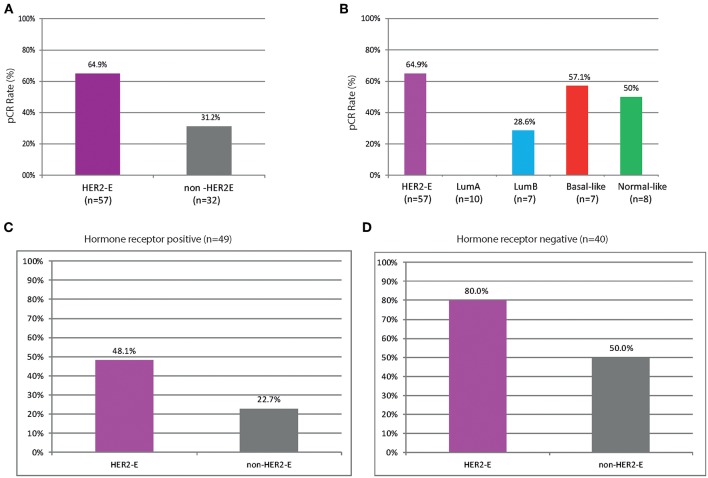
Pathological complete response (pCR) in breast and axilla across the intrinsic subtypes of breast cancer in **(A,B)** the overall cohort; **(C)** Patients with HR-positive disease (*n* = 49); **(D)** Patients with HR-negative disease (*n* = 40). HER2-E, HER2-enriched; non-HER2-E, non-HER2-enriched.

**Figure 4:**
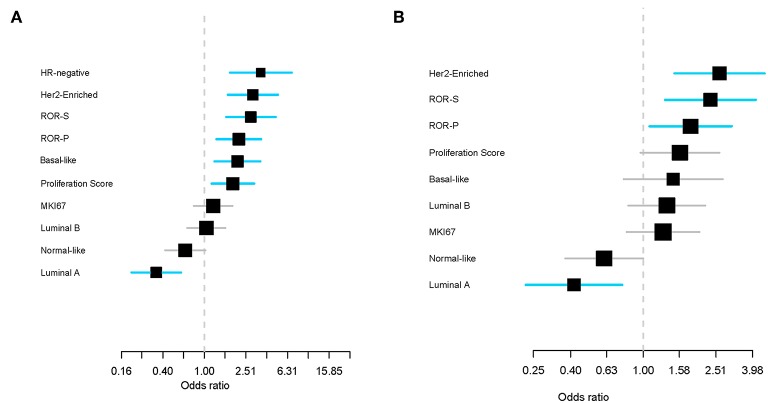
Effect of PAM50 signatures (as continuous variables) on pathological complete response (pCR) in the univariate analysis **(A)** and after adjusting for hormone receptor variables **(B)**. Each signature has been standardized to have a mean of 0 and a standard deviation of 1. The size of the square is inversely proportional to the standard error. Horizontal bars represent the 95% CIs of ORs. Statistically significant variables are shown in blue. Each gene signature has been evaluated individually and ranked ordered based on the estimated OR. ROR-S, risk of recurrence score based on subtype; ROR-P risk of recurrence score based on subtype and proliferation.

Higher rates of pCR were observed in HER2-enriched tumors compared to non-HER2-enriched subtypes (64.9 vs. 31.2%, OR = 4.07, 95% CI 1.65–10.61, *P* < 0.002) regardless of HR status (Figure 3). None of the luminal A samples achieved a pCR and only two samples with luminal B disease (28.6%) achieved a pCR. We evaluated the association between PAM50 signatures, HR status (by IHC), and ki67 (by IHC and by gene expression) with pCR. HR-negative status and five of the eight PAM50 signatures (HER2-enriched, ROR-S based on subtype contents, ROR-P based on subtype contents and proliferation index, Basal-like, and Proliferation score) were significantly associated with pCR, whereas luminal A was associated with non-pCR (*P* < 0.001). HR-negative status, HER2-enriched and luminal A signatures demonstrated the strongest association in predicting pathological response (Figure 4A). After adjusting for HR status, HER2-enriched, ROR-S and ROR-P were significantly associated with pCR and luminal A with non-pCR (Figure 4B).

We then assessed the association between individual expression of 105 genes and pCR. The expression of 14 genes was significantly associated with pCR, including ERBB2, CCNE1, genes involved in cell survival and migration (like FGFR4 and GRB7), and genes related with DNA repair and replication pathway (EXO1, ORC6L, and RRM2). On the contrary, the expression of 21 genes was significantly associated with non-pCR, including BCL2, ESR1, GATA3, KRT19, MYC, PGR, PIK3CA, and SLC39A6 (Supplemental Data).

### Residual Disease and Paired Samples From Patients Not Achieving a pCR

**Figure 5:**
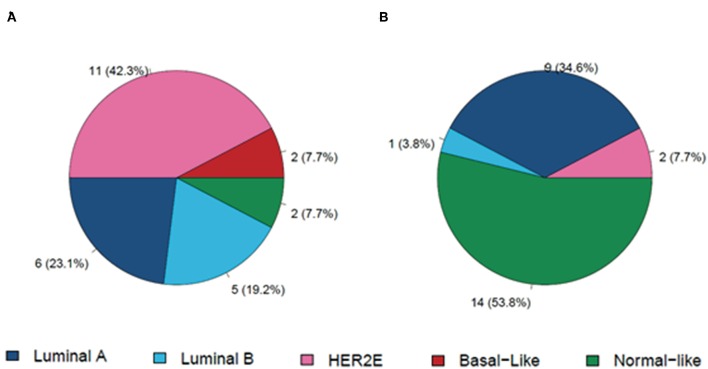
Distribution of molecular subtypes in a cohort of patients with residual disease and paired baseline **(A)** and surgical specimens **(B)**. HER2E, HER2-enriched.

Out of the 66 patients with residual disease at surgery, gene expression was successfully performed in 28 surgical specimens (42.4%). Residual subtype distribution was as follows: normal-like (50.0%), luminal A (32.1%), HER2-enriched (14.3%), and luminal B (3.5%). Of these 28 surgical specimens with residual disease, 26 had pre- and post-treatment paired samples. As expected, the baseline distribution of the intrinsic subtypes in this cohort of patients that did not achieved a pCR, differed from the overall cohort (Figure 5), with less proportion of HER2-enriched subtype (42.3 vs. 64%) and nearly double the proportion of luminal samples (42.3 vs. 19.1%). Regarding changes in intrinsic subtypes in pre- and post-treatment paired samples with residual disease, most of HER2-enriched tumors (81.8%) converted to non-HER2-enriched, whereas 66.7% of luminal A samples maintained the same subtype. Interestingly, in this cohort of paired samples there were 7 conversions to HER2-negative in residual disease (10 cases in the overall cohort).

Next, we analyzed changes in the expression of the 8 PAM50 signatures in those 52 pre- and post-treatment paired samples. Most of them underwent significant changes: a decrease in expression of HER2-enriched and luminal B signatures, proliferation score, ROR-S, and ROR-P PAM50 signatures were observed in most samples, as well as an important increase in luminal A and normal-like signatures. On the contrary, basal-like signatures showed no changes (Figure S3). Regarding single genes, 90 changed significantly, with a false discovery rate of <5%. Thirty-five genes, mostly related to stroma (CAV1, VIM, MET, MMP) were overexpressed in post-treatment samples compared to baseline, whereas 55 genes decreased in expression (Supplemental Data). Most of the downregulated genes in post-treatment samples are involved in functions such as cell cycle and proliferation (EXO1, CENPF, MKI67).

## Discussion

HER2-positive breast cancer is indeed a clinically and biologically heterogeneous disease not fully recapitulated by HR status. In this consecutive series of HER2-positive breast cancer patients treated with trastuzumab-based primary chemotherapy, all the main intrinsic molecular subtypes were identified by gene expression analyses. Intrinsic subtype distribution differed significantly between HR-negative and HR-positive tumors. Importantly, HER2-enriched was the predominant subtype, not only in the overall and HR-negative cohorts (64 and 75%, respectively) but also in the HR-positive subgroup (55%). Tumor heterogeneity within this series of HER2-positive breast cancer modulated response to neoadjuvant treatment. The highest pCR rate was among patients with HER2-enriched tumors, which was more than double the pCR rate of patients with non-HER2-enriched tumors (65 vs. 31%), even in patients with HR-positive tumors (48 vs. 23%).

HER2-enriched subtype has consistently been associated with achieving the highest rate of pCR among HER2-positive tumors (15, 17–21), even in the absence of chemotherapy, with just dual HER2-blockade (21). In the clinical trials that have evaluated efficacy of HER2-targeted agents (e.g., trastuzumab, pertuzumab, and lapatinib) in combination with neoadjuvant chemotherapy or dual blockade alone, the pCR observed among the HER2-enriched subtype varies between 41 and 70%, with the highest rate being achieved with dual-HER2 blockade and chemotherapy. In our study, the pCR rate of the HER2-enriched subgroup was 65%, similar to that achieved in the CALGB study (70%), one of the highest ever described in HER2-positive breast cancer (15), regardless of treatment arm or HR status. It is important to note that, in our study, treatment consisted of single-trastuzumab given concomitantly with anthracycline-and-taxanes-based neoadjuvant chemotherapy. The overall pCR rate of 53% in our series is similar to that achieved in the ACOSOG Z1041 trial (20), a trial designed to compare the pCR rate either in a sequential or concurrent regimen of an anthracycline-and-taxanes-based chemotherapy and trastuzumab (like the one used in our study), which ultimately found no difference between both arms. In this trial, cases classified as HER2-enriched subtype by RNA-seq analysis were also more likely to achieve a pCR compared to non-HER2-enriched tumors.

To our knowledge, our study is the first one to demonstrate the association of five out of eight PAM50 signatures (HER2-enriched, ROR-S, ROR-P, Basal-like, and Proliferation score) with pCR, whereas luminal A signature was associated with non-pCR. Moreover, HR-negative status and HER2-enriched subtype (and signature) demonstrated the strongest association in predicting pCR and luminal A signature with non-pCR. Importantly, intrinsic subtype was an independent, additional predictive factor of pCR to HR status.

Regarding baseline distribution of molecular subtypes, our results are in accordance with previous reports such as the PAMELA trial (21) and the APT trial (26, 27) where the largest subset of baseline samples was classified as HER2-enriched (66.9 and 65%, respectively). In contrast, in the CALGB40601 (15) study and the Cher-LOB trial (18), the proportion of HER2-enriched subtype at baseline was similar to that of luminal A and luminal B (31 and 27%, respectively) and luminal subtypes predominated among HR-positive tumors. This fact could explain why the overall pCR rate in the control arm in the Cher-LOB trial (with the same treatment schema as in our series) was surprisingly low (25%) (18). Another explanation is that the PAMELA trial, the APT trial, and our study all used the nCounter platform, whereas the CALGB40601 study used RNAseq and Cher-LOB used microarrays.

Limited data exist regarding the distribution of molecular subtypes in residual disease after neoadjuvant therapy and in pre- and post-treatment paired samples. In the present study, we examined changes in gene expression and molecular subtype in paired samples of patients with residual disease. A lower proportion of HER2-enriched subtypes and almost twice the number of luminal tumors than in the overall cohort were found at baseline. The most frequent subtype in not eradicated post-treated tumors, excluding normal-like, was the luminal A subtype, as occurred in the CALGB40601 study (15). In the paired samples, most HER2-enriched tumors changed to non-HER2-enriched, whereas most luminal A samples maintained the same subtype. The observed changes in molecular subtype could be attributed to reduced proliferation and/or changes in tumor and stroma cellularity. Residual tumors also showed a substantial modulation of genes, with downregulation of genes involved in proliferation and cell cycle function and upregulation of those related mostly to stroma. However, gene expression analyses cannot distinguish between intra-tumor heterogeneity, stromal alterations or a true treatment effect and may be a mixture of all three. The down regulation of the HER2-enriched, luminal B and proliferation PAM50 signatures (proliferation score, ROR-S, and ROR-P) and the overexpression of the luminal A and normal-like signatures, seen in those paired samples from our study could be explained by peritumoral stromal contamination. We note that these analyses should be interpreted with caution, due to the exploratory nature and small sample size of the study.

This study has several strengths and limitations. It was done in a real-world setting, at a single institution, and it has a long-term follow-up. Patients were homogeneously treated with trastuzumab-based therapy and the study also included evaluation of pre- and post-treatment paired samples. Nevertheless, gene expression did not include immune signatures, which other studies have found to be an independent predictor of response to HER2 targeting beyond PAM50 intrinsic subtypes (15, 18–20), and mutational status (such as PIK3CA) was not analyzed either. Additionally, the current standard neoadjuvant therapy for HER2-positive breast cancer include dual-HER2-blockade with trastuzumab and pertuzumab.

Biologic heterogeneity within HER2-positive breast cancer can determine response to treatment and prognosis as shown in clinical trials, and in everyday clinical practice as shown in our study. Yet, not all HER2-positive breast cancer patients may need to be treated in the same manner. The combination of HER2-targeted therapy alone (dual HER2 blockade with or without endocrine therapy) has shown activity in a substantial percentage of patients, eradicating HER2-positive tumors without chemotherapy and with a favorable toxicity profile (21, 28, 29). However, we need to be able to identify which patients can benefit from this de-escalation strategy and if there is a survival benefit in achieving a pCR with just dual blockade and no chemotherapy. Interestingly, findings from additional exploratory subgroup analyses in the NOAH study (30) showed that the prognostic effect of pCR for event-free survival and overall survival was statistically significant only in patients treated with chemotherapy and trastuzumab and not in patients treated with chemotherapy alone. What does seem clear is that HER2 expression as a single biomarker of treatment response is not enough to develop rational individualized therapeutic regimens. There is an urgent need to find robust predictive biomarkers of response or resistance to the anti-HER2 approach, other than HER2-positivity, in order to individualize treatment and identify different populations of patients who need more treatment or others who may avoid unnecessary treatments and their related toxicities. Serial changes in gene expression, tumor cells or immune cells, as was done in the PAMELA trial (21, 31), may identify early predictive markers of response or resistance than just baseline or residual intrinsic subtypes alone.

## Conclusion

Our data show that, outside of a clinical trial, PAM50 HER2-enriched intrinsic subtype predicts pCR beyond HR status following trastuzumab-based chemotherapy in HER2-positive disease. The clinical value of intrinsic molecular subtype in residual disease warrants further investigation.

## Data Availability

All datasets generated for this study are included in the manuscript and/or the Supplementary Files.

## Ethics Statement

This study was approved by the Institutional Review Board of H.U. Bellvitge, L'Hospitalet (Barcelona), and all patients signed informed consent forms to allow molecular analyses to be performed on their tissue samples.

## Author Contributions

SP and APr: conception and design. SP, APe, FC, CF, IM, AF-O, AS, MR, AG-T, MG-G, and APr: provision of study materials or patients. SP, APe, FC, LP, JP-M, LV, MB, PG, CF, IM, AF-O, AS, MR, MG-G, and APr: collection and assembly of data. SP, APe, FC, LP, JP-M, and APr: data analysis and interpretation. SP, LP, and APr: manuscript writing. All authors: final approval of manuscript and accountable for all aspects of the work.

### Conflict of Interest Statement

SP has received honoraria for talks and travel grants from Roche, outside of the submitted work and has served as an advisor/consultant to Polyphor. CF has received travel grants from Celgene, outside of the submitted work. AS has received honoraria for talks and travel grants from Roche and Eisai, outside of the submitted work. MG-G has received honoraria for talks from Roche, Pfizer, Novartis, and Pierre Fabre and travel grants from Roche and Daiichi-Sankyo all outside of the submitted work. MG-G has served as an advisor/consultant to Pfizer, Novartis, and Daiichi-Sankyo. Advisory role of APr for Nanostring Technologies. The remaining authors declare that the research was conducted in the absence of any commercial or financial relationships that could be construed as a potential conflict of interest.

## Supplementary Material

Figure S1 Flow diagram detailing the study population.

Figure S2 Survival outcomes based on pathological complete response (pCR). Disease-free survival (DFS) by hormone receptor (HR) status **(A)**; Overall survival (OS) by HR status **(B)**; DFS by HER2-Enriched and non-HER2-Enriched **(C)**; OS by HER2-Enriched and non-HER2-Enriched **(D)**.

Figure S3 Changes in the PAM50 signatures in paired samples (i.e., baseline vs. post-treatment) of patients not achieving a pCR.

Table S1 Changes in the expression of the 105 genes between pre-and post-treatment paired samples in patients not achieving a pathologic complete response (pCR).
