# Corrigendum: PAM50 Subtypes in Baseline and Residual Tumors Following Neoadjuvant Trastuzumab-Based Chemotherapy in HER2-Positive Breast Cancer: A Consecutive-Series From a Single Institution

**DOI:** 10.3389/fonc.2019.00967

**Published:** 2019-09-25

**Authors:** Sonia Pernas, Anna Petit, Fina Climent, Laia Paré, J. Perez-Martin, Luz Ventura, Milana Bergamino, Patricia Galván, Catalina Falo, Idoia Morilla, Adela Fernandez-Ortega, Agostina Stradella, Montse Rey, Amparo Garcia-Tejedor, Miguel Gil-Gil, Aleix Prat

**Affiliations:** ^1^Department of Medical Oncology-Breast Cancer Unit, Institut Català d'Oncologia (ICO)-H.U.Bellvitge-Institut d'Investigació Biomèdica de Bellvitge (IDIBELL), Universitat de Barcelona, Barcelona, Spain; ^2^Department of Pathology-Breast Cancer Unit, Institut Català d'Oncologia (ICO)-H.U.Bellvitge-Institut d'Investigació Biomèdica de Bellvitge (IDIBELL), Universitat de Barcelona, Barcelona, Spain; ^3^Department of Medical Oncology, Hospital Clínic de Barcelona, Universitat de Barcelona, Barcelona, Spain; ^4^Clinical Research Unit, Institut Català d'Oncologia (ICO)-L'Hospitalet, Barcelona, Spain; ^5^Department of Pharmacy, Institut Català d'Oncologia (ICO)-L'Hospitalet, Barcelona, Spain; ^6^Department of Gynecology-Breast Cancer Unit, Institut Català d'Oncologia (ICO)-H.U.Bellvitge-Institut d'Investigació Biomèdica de Bellvitge (IDIBELL), Universitat de Barcelona, Barcelona, Spain

**Keywords:** breast cancer, HER2, pathological complete response, gene expression, molecular intrinsic subtype, residual disease, paired samples

In the original article, there was a mistake in Figure 1 as published. The colors of the labels used for Figures 1B,D were incorrect. pCR should be in red and non-pCR should be in blue. The corrected Figure 1 appears below.

**Figure 1:**
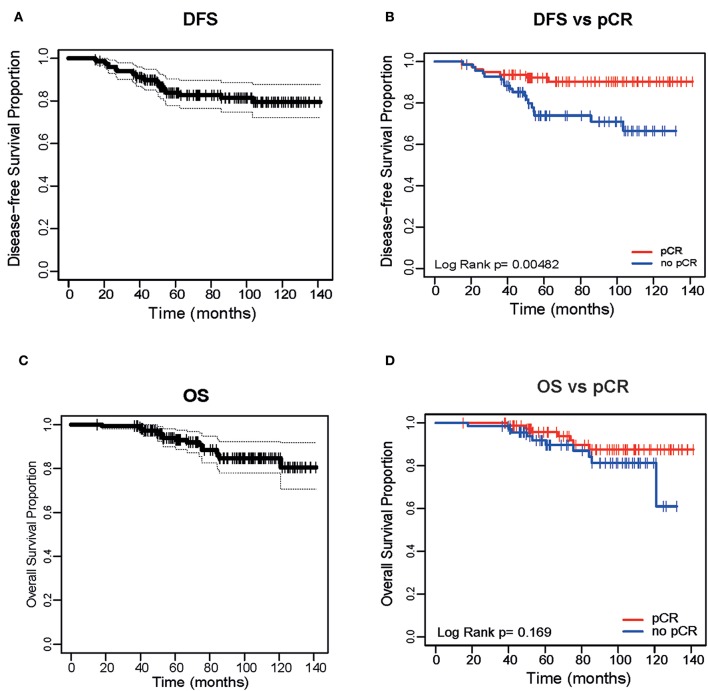
Disease Free Survival of the entire cohort **(A)** and based on pathological complete response (pCR) **(B)**. Overall Survival (OS) of the entire cohort **(C)** and based on pCR **(D)**.

The authors apologize for this error and state that this does not change the scientific conclusions of the article in any way. The original article has been updated.

