# Supplementary figures and images for: PAM50 Subtypes in Baseline and Residual Tumors Following Neoadjuvant Trastuzumab-Based Chemotherapy in HER2-Positive Breast Cancer: A Consecutive-Series From a Single Institution
Source: Front Oncol. 2019 Aug 6;9:707. doi: 10.3389/fonc.2019.00707 (PMC6691353; doi:10.3389/fonc.2019.00707)

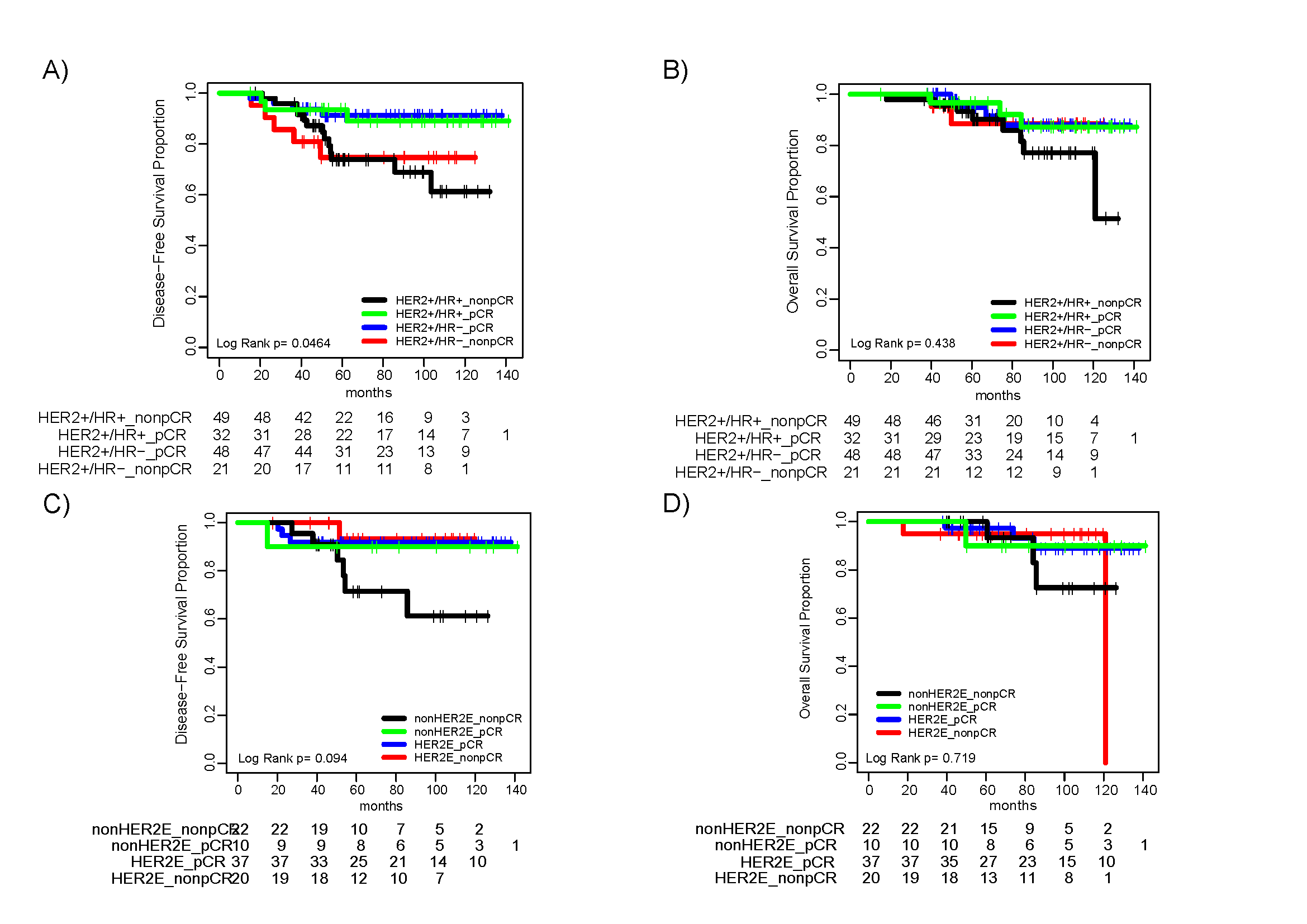

Supplement: Figure S2 — Survival outcomes based on pathological complete response (pCR). Disease-free survival (DFS) by hormone receptor (HR) status (A); Overall survival (OS) by HR status (B); DFS by HER2-Enriched and non-HER2-Enriched (C); OS by HER2-Enriched and non-HER2-Enriched (D). [file Image_2.TIFF]

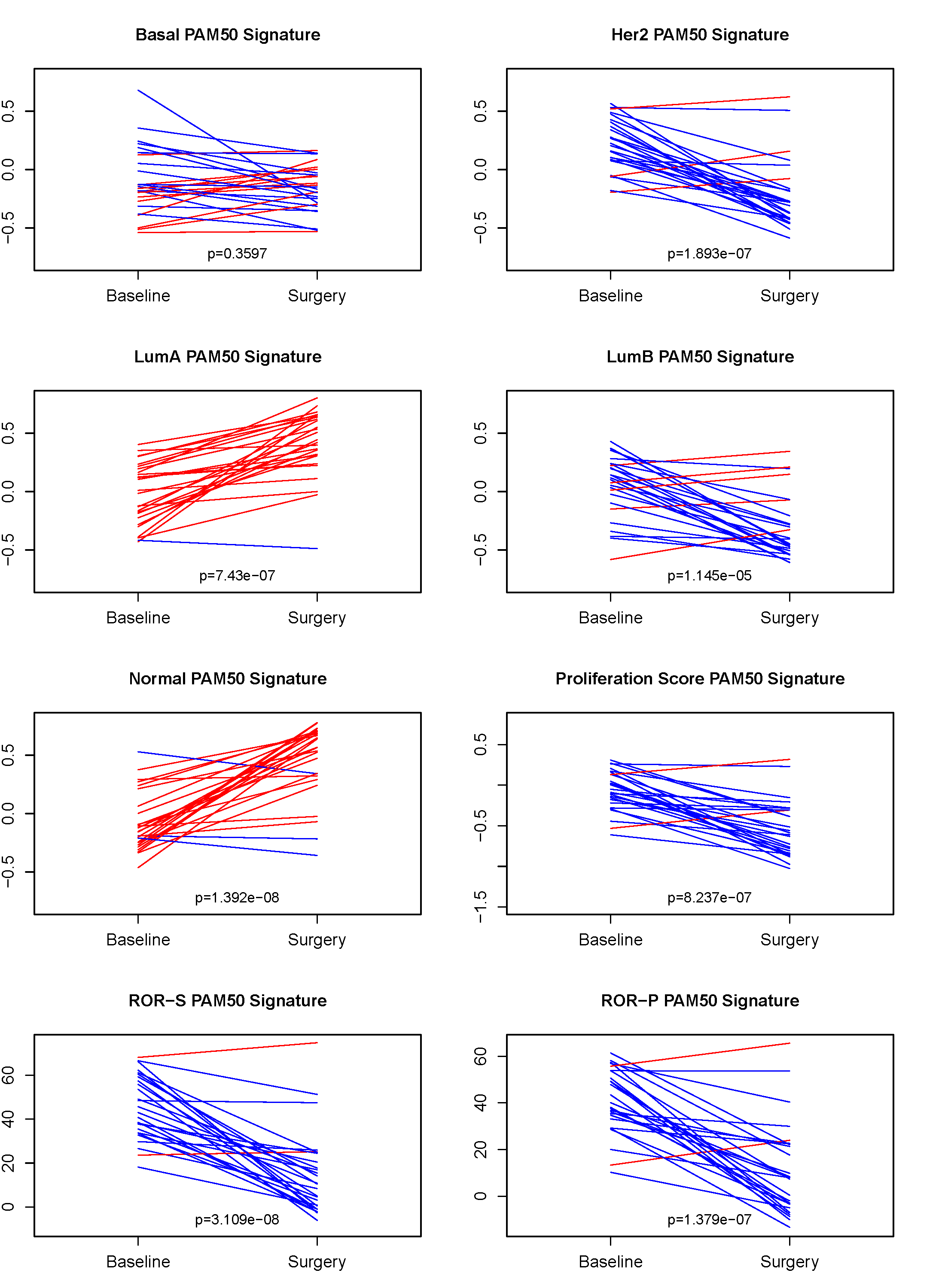

Supplement: Figure S3 — Changes in the PAM50 signatures in paired samples (i.e., baseline vs. post-treatment) of patients not achieving a pCR. [file Image_3.TIFF]
